# A Elevada Pressão do Combate a Pandemia da COVID-19

**DOI:** 10.36660/abc.20210811

**Published:** 2021-11-01

**Affiliations:** 1 Instituto do Câncer do Estado de São Paulo São Paulo SP Brasil Instituto do Câncer do Estado de São Paulo, São Paulo, SP - Brasil; 2 Hospital Sírio Libanês São Paulo SP Brasil Hospital Sírio Libanês, São Paulo, SP - Brasil; 3 Hospital Vila Nova Star - Rede D’Or São Paulo SP Brasil Hospital Vila Nova Star - Rede D’Or, São Paulo, SP - Brasil

**Keywords:** COVID-19, Betacoronavírus, Pandemia, Doenças Cardiovasculares, Hipertensão, Idosos, Hospitalização, Comorbidades

Apesar de já estarmos há quase dois anos no enfrentamento dos desafios impostos pelo novo coronavírus, ainda nos resta um longo caminho a ser percorrido. A rápida e fácil propagação do vírus não só preocupa a população e a sociedade científica como um todo, como também escancarou a fragilidade do sistema público de saúde brasileiro, tido como universal. Os números falam por si - as taxas exorbitantes de infectados e, consequentemente, de números de óbitos nos fizeram e fazem questionar a condução desta crise sanitária.

Neste cenário, indivíduos idosos e com comorbidades, tais como hipertensão arterial, diabetes, obesidade e doença arterial coronariana, foram os que mais sofreram com a COVID-19.^1^ O vírus no organismo leva a extensa disfunção endotelial,^2^ intermediada por citocinas inflamatórias e fatores trombogênicos, lesões microvasculares disseminadas e graves complicações, tais como: embolia pulmonar e sistêmica, injúria miocárdica e disfunção renal.^3^ Essas manifestações mostraram-se potencialmente fatais, em especial neste grupo aqui elencado, pela concomitante presença de doenças cardiovasculares (DCV).

Dentre as DCV, a hipertensão arterial (HA) destaca-se por sua elevada prevalência; no Brasil e na China, por exemplo, mais de 20% da população total é hipertensa, valor que chega a 71,7% em indivíduos acima de 70 anos.^4,5^ A HA se mostra como grave problema de saúde pública, sendo a si atribuídos 14% das internações gerais e a responsabilidade por elevado e em ascensão número de óbitos. Em 2015, no Brasil, foram registradas 47.288 mortes por HA, número este que aumentou para 53.022 em 2019.^6^

Deng et al.,^5^ nesta publicação desta edição dos Arquivos Brasileiros de Cardiologia, de modo muito objetivo, avaliaram a associação entre HA e a gravidade/mortalidade em pacientes hospitalizados pela COVID-19 na China. Em uma coorte retrospectiva de 337 pacientes, elencam-se as características clínicas e laboratoriais dos 112 pacientes hipertensos, comparados a um grupo de normotensos. No grupo dos hipertensos, observou-se que eles eram mais idosos, com mais comorbidades associadas (como, doença renal e doença cerebrovascular) e desenvolveram mais complicações no curso da infecção, com maior necessidade de suplementação de oxigênio e evolução para síndrome do desconforto respiratório agudo grave.^5^

De modo consistente, os dados apresentados por estes autores mostram que as provas inflamatórias, como proteína C reativa e procalcitonina estão mais elevadas nestes pacientes, bem como ocorre maiores níveis séricos de marcadores de lesão cardíaca (troponina T, creatina quinase MB e NT-proBNP). O grau de hipertensão arterial também esteve associado a maior gravidade da COVID-19, uma vez que 60% dos pacientes com HA estágio III com COVID-19 desenvolveram quadros críticos da doença.^5^ O estudo também mostrou que HA esteve associada a quase 2,2 mais chances de morrer por COVID-19 (OR: 2,093 [IC95%: 1,094-4,006], p=0,024).^5^

Outros pesquisadores corroboram os achados apresentados por estes autores e também sugerem que a HA é a comorbidade mais comumente relacionada ao aumento de mortalidade em pacientes com COVID-19.^7^ O que explicaria a gravidade associada entre este binômio ainda não foi completamente elucidado. Aventa-se a hipótese de uma possível interface entre o vírus e HA com o sistema renina angiotensina aldosterona,^8^ além da disfunção endotelial que é inerente às duas doenças. A invasão celular pelo vírus seria facilitada pela enzima conversora da angiotensina 2 (ECA-2), que é amplamente encontrada nas células cardíacas e pulmonares. Assim, esse passaporte seria o pontapé inicial para, posteriormente, o vírus desencadear uma exuberante cascata inflamatória e explicando, em parte, o grave acometimento cardiopulmonar imposto pela COVID-19.^9^

De fato, é certo que o combate à pandemia da COVID-19 envolve medidas de isolamento social e vacinação em massa. Contudo, ações que promovam assistência de saúde adequada à população devem ser igualmente priorizadas e mantidas de forma perene no enfrentamento de qualquer contexto adverso, ainda mais evidente neste momento que vivemos. Dessa forma, oportunizar o correto tratamento de doenças tão prevalentes, como a HA, pode contribuir de forma significativa para a redução da mortalidade da COVID-19.
