# Supplementary material for: A Elevada Pressão do Combate a Pandemia da COVID-19
Source: Arq Bras Cardiol. 2021 Nov 1;117(5):922–3. [Article in Portuguese] doi: 10.36660/abc.20210811 (PMC8682096; doi:10.36660/abc.20210811)
Supplement: Translation

# Translation: The High Pressure of Fighting the COVID-19 Pandemic

**Affiliations:** 1 São Paulo SP Brazil Instituto do Câncer do Estado de São Paulo, São Paulo, SP - Brazil; 2 São Paulo SP Brazil Hospital Sírio Libanês, São Paulo, SP - Brazil; 3 São Paulo SP Brazil Hospital Vila Nova Star - Rede D’Or, São Paulo, SP - Brazil

**Keywords:** COVID-19, Betacoronavirus, Pandemics, Cardiovascular Diseases, Hypertension, Aged, Hospitalization, Comorbidities

Although we have been facing the challenges posed by the new coronavirus for almost two years, we still have a long way to go. The rapid and easy spread of the virus not only worries the population and scientific society as a whole but also exposes the fragility of the Brazilian public health system. The numbers speak for themselves - the exorbitant rates of infected people and, consequently, the number of deaths made us question the handling of this health crisis.

In this scenario, elderly individuals with comorbidities such as hypertension, diabetes, obesity and coronary artery disease were the ones who suffered most from COVID-19.^1^ The virus in the body leads to extensive endothelial dysfunction,^2^ mediated by inflammatory cytokines and thrombogenic factors, disseminated microvascular lesions and serious complications, such as pulmonary and systemic embolism, myocardial injury and renal dysfunction.^3^ These manifestations proved to be potentially fatal, especially in the group listed here, mainly due to the concomitant presence of cardiovascular diseases (CVD).

Among CVDs, arterial hypertension (AH) stands out for its high prevalence; in Brazil and China, more than 20% of the total population is hypertensive, a figure that reaches 71.7% in individuals over 70 years of age.^4,5^ Thus, AH appears as a severe public health problem, 14% of general admissions attributed to it, and it is responsible for the high and rising number of deaths. In 2015, in Brazil, 47.288 deaths from AH were recorded, increasing to 53.022 in 2019.^6^

Deng et al.,^5^ in the publication of this issue of the Brazilian Archives of Cardiology, very objectively evaluated the association between AH and severity/mortality in hospitalized patients by COVID-19 in China. In a retrospective cohort of 337 patients, the clinical and laboratory characteristics of 112 hypertensive patients are listed compared to a group of normotensive patients. In the hypertensive group, it was observed that they were older, with more associated comorbidities (such as kidney disease and cerebrovascular disease) and developed more complications in the course of the infection, with a greater need for oxygen supplementation and progression to severe acute distress syndrome.^5^

Consistently, the data presented by these authors show that inflammatory tests such as C-reactive protein and procalcitonin are higher in these patients and higher serum levels of cardiac injury markers (T troponin, creatine kinase MB and NT-proBNP). The degree of arterial hypertension was also associated with greater severity of COVID-19 since 60% of patients with stage III hypertension with COVID-19 developed critical conditions of the disease.^5^ The study also showed that AH was associated with almost 2.2 more chances of dying from COVID-19 (OR: 2.093 [CI95%: 1.094-4.006], p=0.024).^5^

Other researchers corroborate the findings presented by these authors and also suggest that AH is the most commonly associated comorbidity with increased mortality in patients with COVID-19.^7^ What explains the associated severity between this binomial has not yet been fully elucidated. The hypothesis of a possible interface between the virus and AH with the renin-angiotensin-aldosterone system is suggested,^8^ in addition to the endothelial dysfunction that is inherent to both. Cellular invasion by the virus would be facilitated by the angiotensin-2 converting enzyme (ACE-2), which is widely found in cardiac and lung cells. Thus, this passport would be the kick-off for the virus, later, to trigger an exuberant inflammatory cascade, explaining, in part, the severe cardiopulmonary impairment imposed by COVID-19.^9^

In fact, combating the COVID-19 pandemic indeed involves measures of social isolation and mass vaccination. However, actions that promote adequate health care to the population must be equally prioritized and maintained perennially in facing any adverse context, even more evident in this moment in which we live. Thus, providing opportunities for the correct treatment of such prevalent diseases, such as AH, can significantly contribute to the reduction of mortality in COVID-19.
